# Assessment of *EGFR* Gene Expression Following Vitrification of 2-cell and Blastocyst Mouse Embryos

**Published:** 2018

**Authors:** Rouhollah Gazor, Mozhgan Eskandari, Alireza Sharafshah, Mohammad Hadi Bahadori, Mohammad Ghasem Golmohammadi, Parvaneh Keshavarz

**Affiliations:** 1. Department of Anatomy and Cell Biology, Faculty of Medicine, Guilan University of Medical Sciences, Rasht, Iran; 2. Department of Anatomy, Ardabil University of Medical Sciences, Ardabil, Iran; 3. Cellular and Molecular Research Center, Faculty of Medicine, Guilan University of Medical Sciences, Rasht, Iran

**Keywords:** Blastocyst, Cryopreservation, Embryo, Gene expression, Vitrification

## Abstract

**Background:**

Exact mechanisms of fetal harm following vitrification are still unknown. This study was conducted to evaluate the cryopreservation impact on the expression of Epidermal *Growth Factor Receptor (EGFR)* gene in mouse 2-cell and blastocysts.

**Methods:**

To stimulate ovulation in mice, hCG was injected, followed by collecting 2-cells and blastocysts after 44–46 and 88–89 *hr*, respectively. These embryos were divided into two case and control groups. The fresh case group was cryopreserved using cryotop and warmed after 4 mounts. Normal 2-cells were selected based on their morphology and their RNA was extracted. Quantitative expression of *EGFR* gene in both groups was investigated by applying real time-PCR.

**Results:**

The statistical Real-Time (RT)-PCR analyses performed using SPSS revealed that the expression level of *EGFR* gene was diminished in the case group compared to the control group.

**Conclusion:**

The current study indicated the negative effect of cryopreservation on expression amount of *EGFR* gene in 2-cell and blastocyst mouse embryos.

## Introduction

According to the recent studies of World Health Organization (WHO), approximately 10% of couples are infertile worldwide^1^. Molecular control of pre-implantation of mammalian embryos remains largely unknown mainly due to barriers to obtain sufficient quantities of embryos for experiments. Evidence in various fields including transcription inhibition at 1-cell stage, protein synthesis inhibition and all subsequent stages of development after the first cleavage, and the synthesis of all types of RNA at the 2-cell stage have represented the primary activities of the embryonic genome^2^. There is a risk of multiple pregnancies in most IVF programs. In addition, since the factors stimulating the uterus cycles may jeopardize implantation, it is necessary to improve freezing processes.

Since freezing is very time-consuming and not satisfying, most embryologists are trying to find other freezing protocols such as vitrification. Vitrification is a fast and cheap technique applied for freezing embryos (mammalian species) in various stages of development^3^, with unclear genetic effects. It is not yet known whether warmed embryos that are morphologically normal are also genetically normal.

Epidermal Growth Factor Receptor (EGFR) plays an important role in stimulating embryonic cell proliferation and has a critical role in early pregnancy of mammals leading to oocyte maturation, embryo pre-implantation, proliferation of embryo, and trophoblastic cells diffusion into the endometrium. Considering the importance of vitrification process in the survival of live embryos as well as the role of *EGFR* in implantation, this study was conducted to assess the effects of vitrification on *EGFR* (MGI:95294) expression.

## Materials and Methods

80 NMRI female mice aged 4 to 8 weeks were superovulated by an intraperitoneal injection of 5.5 *IU/ml* pregnant mare’s serum gonadotrophin (PMSG; Intervet Folligon 5000 *IU*; Holland) followed 48 *hr* later by 5.5 *IU/ml* hCG (Organon 500 *IU*; Holland).

The study consisted of control (non-vitrified) and case (vitrified-warmed) including 2-cell and blastocyst embryo groups. In the control group, 2-cell embryos were extracted from the pregnant mice’s oviduct after 48 *hr* and blastocysts after 88–89 *hr* after mating. In the case group, the retrieved 2-cell and blastocyst embryos were vitrified and kept for 4 months in liquid nitrogen. Six months after the first freezing, 2-cells and blastocysts (case groups) embedded in nitrogen liquid were warmed. After 2–3 *hr*, RNA extraction was immediately done using kit. Analyses were performed on fresh 2-cell (control group) compared to vitrified 2-cell (case group) embryos and fresh blastocysts (controls) were compared with vitrified blastocysts (cases) for the expressions of *EGFR* gene in two control and case groups.

The RT-PCR was used to assess the quantitative expression of *EGFR* gene transcripts. Primers (5′-GGGATTCTTTCACGCGCACTCCT-3′ as forward and 5′-TTCAGGCCAACGACCGCCAAA-3′ as reverse) used for both real-time and RT-PCR were designed using a primer design software: Primer3 (Whitehead Institute for Biomedical Research)^4^. The SYBER Green PCR kit (Qiagen, Germany) was used to extract RNA from all 80 vitrified and non-vitrified 2-cell and blastocyst embryos. Three cryotops were pooled for every extraction which contained nearly 18 to 20 embryos. Synthesis of cDNA was done through reverse transcriptase. Amplification was followed by a melting curve analysis to confirm PCR product specificity according to the manufacturer’s instructions, followed by assessing PCR product size by gel electrophoresis. For each group, the samples were examined in two independent experiments. These samples were run in duplicate and the mean C_t_-value of each duplicate was used for further calculations.

The mathematical model used in this software is based on the correction for exact PCR efficiencies and the mean Crossing Point (CP) deviation between case and control group. The housekeeping gene was β-actin as a reference gene used for normalization of data. The expression ratio results of the investigated transcripts were tested for significance by PairWise Fixed Real-location Randomization Test^©^^5^ and plotted using Standard Error (SE) estimation *via* a complex Taylor algorithm using the REST-MCS Software (http://rest.gene-quantification.info/). *EGFR* gene expression level in normal blastocysts and 2-cell embryos of the vitrified-warmed group and control groups was tested for statistical significance using the Chi-square (*χ*^2^) test by SPSS program (SPSS 11.5; Chicago, IL, USA). The significance level was assigned at p<0.05.

## Results

**Figure 1.**
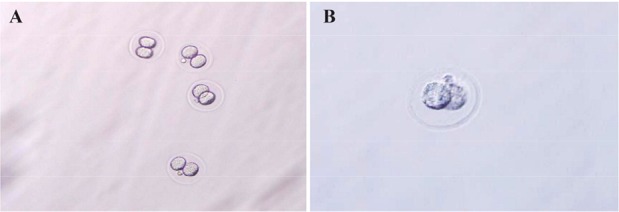
2-cell embryo (A) before freezing and (B) while freezing (magnified ×100). 337.

**Figure 2.**
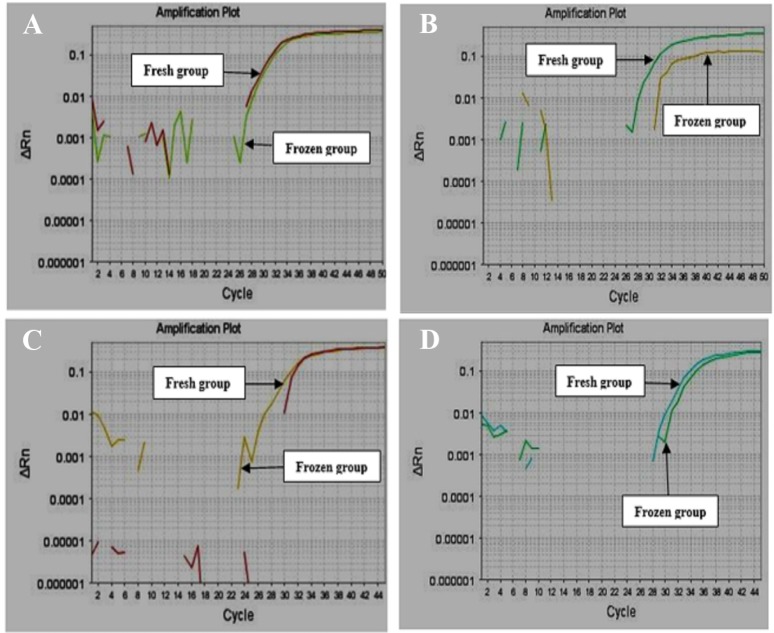
A) β-actin and B) *EGFR* amplification curves in 2-cell group. C) β-actin and D) 338 *EGFR* amplification curves in blastocyst group.

Control group including 2-cell embryos before freezing and case group including 2-cell embryos after freezing by vitrification procedure are shown in figure 1. The results indicated that the specific segments of β-actin and *EGFR* had a warming peak at 75.1 and 79.9, respectively. Amplification curves of β-actin gene indicated that exponential amplification initiated after cycle 25. There was no difference between reactions of fresh and frozen groups in both 2-cell and blastocyst embryos. *EGFR* gene amplification occurred after cycle 28; accordingly, the exponential amplification of *EGFR* gene started at higher cycles for the reaction of frozen samples in comparison with fresh samples in two embryo cell groups (Figure 2). The 2^−ΔΔCt^ procedure was applied for determining the alterations of expression and *EGFR* was selected as the target gene. *EGFR* gene expression increased 1.53 times in frozen and warmed 2-cell embryos compared to control 2-cell samples (Table 1). Furthermore, studying expression changes in *EGFR* gene in frozen and warmed blasto-cyst embryos demonstrated a 1.54 fold increase compared with control blastocyst embryos without freezing and warming (Table 2).

**Table 1.** Data analysis results of *EGFR* expression in 2-cell group

**2-cell control**		**2-cell frozen**	

***EGFR***	**β actin**	***EGFR***	**β actin**
30.7	29.95	33.46	32.28
32.2	31.28	30.8	30.62
31.8	30.61	31.1	31.46
94.7/3=31.56±0.77	91.84/3=30.61±0.66	95.36/3=31.78±1.45	94.36/3= 31.45±0.83
ΔCT2=31.56−30.61=0.95		ΔCT1=31.78−31.45=0.33	
ΔΔCT= 0.33–0.95=−0.62 2^−ΔΔCt^ = 2^0.62^ = 1.53			

*EGFR* gene expression changes in samples which were frozen and thawed with a 1.53 time increase in 2-cell frozen group in comparison with control samples.

**Table 2.** Data analysis results of *EGFR* expression in blastocyst group

**Blastocyst (control)**		**Blastocyst (frozen)**	

***EGFR***	**β actin**	***EGFR***	**β actin**
33.92	30.9	32.24	32.06
30.2	30.8	32.6	33.9
31.34	31.6	31.8	30.4
95.46/3=31.82±1.9	93.3/3=31. 1±0.43	96.64/3=32.21±0.4	96.36/3=32.12±2.47
ΔCT2=31.82−31.1=0.72		ΔCT1=31.21–32.12=0.09	
ΔΔCT= 0.09–0.72=−0.63 2^−ΔΔCt^ = 2^0.63^ = 1.54				

Frozen blastocyst embryos group in comparison with control group showed an increasing number of 1.54 times in *EGFR* gene expression.

## Discussion

The results of the present study showed that freezing reduces the expression of *EGFR* in both 2-cell and blastocyst experiment groups. Riesco *et al* achieved similar results, suggesting that gene expression might change during freezing and warming. To explain this phenomenon, they proposed that cryopreservation affects the stability of mRNA and thus some of them are susceptible to degradation. According to their study, regulation of some mRNAs not only inhibits translation of mRNAs but also involves an alteration in the length of polyA tail. Therefore, the efficiency of translation and stability of mRNA is affected^6^. Qu *et al* investigated the expression of TGFα and EGF receptors in follicles of human ovarian tissue before and after freezing and found that freezing does not change immunologic reaction to TGFα, EGF, and EGFR^7^. This finding is inconsistent with ours and needs further investigation. Tachataki *et al* investigated TSC2 expression in frozen human embryos and concluded that freezing alters the normal pattern of gene expression during preimplantation development^8^. This result is consistent with our findings; i.e. the reduced gene expression by vitrification.

## Conclusion

In conclusion, our results showed that vitrification reduces expression of *EGFR* in embryos in 2-cell and blastocyst stages.
